# Direct observation of spreading precursor liquids in a corner

**DOI:** 10.1093/nsr/nwad119

**Published:** 2023-05-05

**Authors:** Weining Miao, Shihao Tian, Quanzi Yuan, Ye Tian, Lei Jiang

**Affiliations:** Key Laboratory of Bioinspired Smart Interfacial Science, Technical Institute of Physics and Chemistry, Chinese Academy of Sciences, Beijing 100190, China; School of Future Technology, University of Chinese Academy of Sciences, Beijing 100049, China; State Key Laboratory of Nonlinear Mechanics, Institute of Mechanics, Chinese Academy of Sciences, Beijing 100190, China; School of Engineering Science, University of Chinese Academy of Sciences, Beijing 100049, China; State Key Laboratory of Nonlinear Mechanics, Institute of Mechanics, Chinese Academy of Sciences, Beijing 100190, China; School of Engineering Science, University of Chinese Academy of Sciences, Beijing 100049, China; Key Laboratory of Bioinspired Smart Interfacial Science, Technical Institute of Physics and Chemistry, Chinese Academy of Sciences, Beijing 100190, China; School of Future Technology, University of Chinese Academy of Sciences, Beijing 100049, China; Key Laboratory of Bioinspired Smart Interfacial Science, Technical Institute of Physics and Chemistry, Chinese Academy of Sciences, Beijing 100190, China; School of Future Technology, University of Chinese Academy of Sciences, Beijing 100049, China

**Keywords:** precursor liquid, spreading spatial–temporal characteristic, direct observation, corner, ionic liquids

## Abstract

Precursor liquid is a nanoscale liquid creeping ahead of the macroscopic edge of spreading liquids, whose behaviors tightly correlate with the three-phase reaction efficiency and patterning accuracy. However, the important spatial–temporal characteristic of the precursor liquid still remains obscure because its real-time spreading process has not been directly observed. Here, we report that the spreading ionic liquid precursors in a silicon corner can be directly captured on video using *in situ* scanning electron microscopy. *In situ* spreading videos show that the precursor liquid spreads linearly over time (${\rm{\Delta }}L\ \sim\ {\rm{\Delta }}T$) rather than obeying the classic Lucas–Washburn law ($l\ \sim\ {t}^{1/2}$) and possesses a characteristic width of ∼250–310 nm. Theoretical analyses and molecular dynamics simulations demonstrate that the unique behaviors of precursor liquids originate from the competing effect of van der Waals force and surface energy. These findings provide avenues for directly observing liquid/solid interfacial phenomena on a microscopic level.

## Teaser

The spreading ionic liquids precursor was directly captured on video in a silicon corner with in-situ scanning electron microscopy, and was found to spread linearly over time.

## INTRODUCTION

Liquid wetting is ubiquitous and critical in fields ranging from chemical reactions [1], separation [2] and material fabrication [3] to power generation [4]. During liquid spreading, a nanoscale precursor liquid will be developed ahead of the macroscopic liquid droplet, which was predicted to exist a centenary ago and later probed [5–7]. The intrinsic characteristic of precursor liquid is closely related to the performance of nanofluids [8,9], three-phase reactions [10], surface patterning [11,12] and so on. Investigating its dynamic behaviors can enormously improve our understanding of the underlying physical mechanisms during the liquid-wetting process [13] and contribute to the design of high-performance structures.

In the past, diverse techniques, including ellipsometry [14,15], interferometry [16], atomic force microscopy [17] and transmission electron microscopy [18] have been applied to probe the profiles of quasi-static precursor liquids and various precursor liquids have been measured, such as the ∼100-nm-thick silicone oil precursor liquid on silicon [16] and the ∼10-nm-thick ionic liquids precursor on nanowire [18]. However, it is still a formidable challenge to track the frontier position of the spreading precursor liquid in real time and capture its spatial–temporal characteristic during spreading, limited by a bottleneck inherent in these techniques—that is, each measurement takes a long time while the precursor liquid is also in motion. Extensive efforts have been paid by researchers through simulations or theoretical analyses to forecast the possible dynamic behavior of precursor liquid and various hypotheses have been proposed, such as spreading linearly over time [19], spreading with the square root of time [20] and so on [21]. However, because there is no evidence of direct observation, the spatial–temporal characteristic of precursor liquids has remained controversial for nearly a century.

Here, we report that the dynamic spreading process of ionic liquid precursors can be directly observed in a silicon corner using *in situ* scanning electron microscopy (SEM) and further demonstrate that the ionic liquids precursor follows a universal spatial–temporal characteristic of that spreading linearly over time (${\rm{\Delta }}L\ \sim\ {\rm{\Delta }}T$), which is independent of the properties of ionic liquids themselves. Across detailed investigation and theoretical analyses, we find that the precursor liquid possesses a characteristic width of ∼250–310 nm, which results from the competing effect of van der Waals force and surface energy. Molecular dynamics simulations further demonstrate that the spatial–temporal characteristic of precursor liquids is universal for corners with different opening angles ($2\alpha $). Accordingly, a complete model depicting the liquid dynamic spreading process in a corner is established. Our findings not only provide insights into the microscopic wetting process but also would provide inspiration for directly observing other interfacial phenomena on a microscopic level.

## RESULTS AND DISCUSSION

### Model of spreading liquids in corner

**Figure 1.**
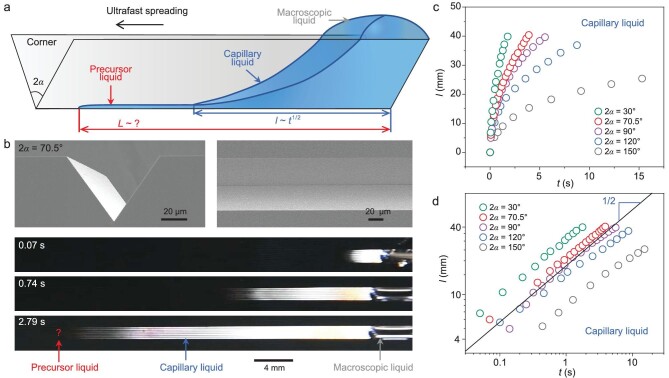
Spreading liquids in a corner. (a) Model of the spreading liquids in a corner with an opening angle of 2*α*, which is divided into three regions, including macroscopic liquid, capillary liquid and precursor liquid. The spreading length of the capillary liquid follows a power law of $l\sim{t}^{1/2}$. The spatial–temporal characteristic of the precursor liquid is still obscure. (b) SEM images of the silicon corner with a 2α of 70.5° and the sequences of the ultrafast spreading process of 2 μL of water in the silicon corner. (c) Real-time spatial–temporal evolution curves of the spreading capillary liquids in corners with different 2*α* values. The liquid spreading speed increases as 2*α* decreases. (d) Logarithmic plot of the spreading length of capillary liquid versus time. The solid line indicates a power law of 1/2. *l* and *t* in (c) and (d) represent the spreading length and time during the entire spreading process from dripping the water droplet, respectively.

Corner [22,23], a structure widely utilized in nature to manipulate liquids, such as ultrafast liquid spreading [24] and directional liquid transport [25,26], provides us with an inspired platform on which to investigate the dynamic behavior of precursor liquids in real time. The spreading liquids in a corner can be divided into three regions (Fig. 1a), including the macroscopic liquid region above the corner plane, the capillary liquid region and the nanoscale precursor liquid region [27].

The silicon corner, which is composed of two intersecting smooth silicon (111) crystal faces formed by anisotropic etching of potassium hydroxide on a <100> silicon wafer [28], was chosen as the substrate to observe the precursor liquid spreading process and illustrate the model because it is sharp and conductive (Fig. 1b). $2\alpha $ of the silicon corner is 70.5°. As the water-spreading video frames captured from Supplementary Video S1 (Fig. 1b) show, the capillary liquid region and the macroscopic liquid region can be clearly identified through optical observation. As $2\alpha $ decreases, the capillary liquids spread faster (Fig. 1c and Supplementary Fig. S1) and all obey the classic Lucas–Washburn law, $l\sim{t}^{1/2}$ (Fig. 1d), which is a universal characteristic to describe the dynamics of capillary rise [29,30]. However, in the model, the dynamic spreading process of precursor liquid has not been directly observed and we hypothesized that the precursor liquid in the corner possesses a comparable and universal characteristic (Fig. 1a).

### *In situ* observation of spreading ionic liquids precursor

We tried to directly observe the dynamic spreading process of precursor liquid utilizing *in situ* SEM. Ionic liquids were selected as the spreading liquids due to their ultralow vapor pressures that facilitate *in situ* SEM imaging under a high vacuum condition [31,32]. The intrinsic contact angles (CAs) of the used ionic liquids on the silicon were small, which satisfies the Concus–Finn condition [33] (${\rm{CA}} < \frac{\pi }{2}-\alpha \ = \ 54.75^\circ $) for spreading in the silicon corner (Supplementary Table S1). Besides, it should be noted that the spreading law of the capillary region in corners is universal for both ionic liquids and water (Supplementary Fig. S2), hence ionic liquids can be utilized as typical liquids to investigate the intrinsic characteristic of precursor liquid in the corner.

**Figure 2.**
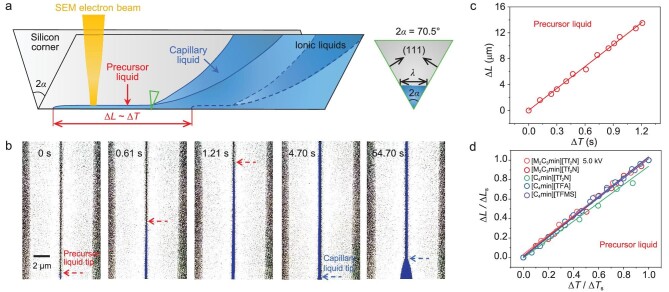
*In situ* spreading process of ionic liquid precursors in a corner. (a) Schematic illustrating the *in situ* SEM imaging method to record the spreading process of ionic liquid precursors in a silicon corner. The spreading precursor liquid follows a law of ${\rm{\Delta }}L\ \sim\ {\rm{\Delta }}T$. The right panel represents the enlarged cross-section of the green triangle position in the left panel. The top width of the precursor liquid is defined as its characteristic width (*λ*). (b) SEM image frames captured from Supplementary Video 2 at 10.0 kV, illustrating the spreading process of 1,2-dimethyl-3-propylimidazolium bis(trifluoromethanesulfonyl)imide [M_2_C_3_min][Tf_2_N] precursor liquid in the corner. The liquids are pseudo-colored in blue. The forefront positions of the precursor liquid and capillary liquid are marked with red and blue dashed arrows, respectively. (c) Real-time spatial–temporal plot of the spreading [M_2_C_3_min][Tf_2_N] precursor liquid. The precursor liquid spreads linearly over time. ${\rm{\Delta }}L$ and ${\rm{\Delta }}T$ represent the changes in length and time of the precursor liquid after it enters the field of view of *in situ* SEM from 0 s, respectively. (d) Dimensionless spatial–temporal plot of the spreading precursor liquids of four ionic liquids at 10.0 kV and [M_2_C_3_min][Tf_2_N] at 5.0 kV. Δ*L*_s_ and Δ*T*_s_ represent the changes in length and time within the statistical period, respectively.

After dripping a 1-μL ionic liquid droplet on one end of a 2 cm × 2 cm silicon wafer containing parallel corners with a top width of 7.6 μm and a spacing of 18.0 μm (Supplementary Fig. S3), the whole sample device was loaded into the Hitachi SU8010 SEM with a secondary electron image resolution of 1.0 nm at an acceleration voltage of 10.0 kV and the electron beam was focused at the forefront of the spreading ionic liquids to record its spreading process (Fig. 2a and Supplementary Fig. S4). A typical spreading process of 1,2-dimethyl-3-propylimidazolium bis(trifluoromethanesulfonyl)imide [M_2_C_3_min][Tf_2_N] in the corner (accelerating voltage: 10.0 kV; current: 10.0 μA) is shown in Supplementary Video S2, which contains the precursor liquid spreading in the front and the capillary liquid following behind. As the time-resolved investigation of the [M_2_C_3_min][Tf_2_N] precursor liquid spreading process is shown in Fig. 2b (Supplementary Video S2, from 0 s to 1.21 s), the precursor liquid possesses a stable width when spreading forward along the corner tip, and the leading edge (marked with dashed red arrow) can be clearly identified. After 1.26 s, the frontier of the precursor liquid spread beyond the field of view of *in situ* SEM. From 1.26 to 4.70 s, the precursor liquid was also spreading forward, although its boundary and width did not appear to change. After 4.7 s, the capillary liquid behind the precursor liquid spread into the field of view of *in situ* SEM and the forefront position of the capillary liquid was marked with a blue dashed arrow (Fig. 2b). After measuring the spreading distances of the precursor liquid, its corresponding real-time spatial–temporal characteristic can be obtained (Fig. 2c). Surprisingly, it is elucidated that the spreading length of the precursor liquid increases with the first power of time (${\rm{\Delta }}L\ \sim\ {\rm{\Delta }}T$) rather than obeying the classic Lucas–Washburn law ($l\sim{t}^{1/2}$).

To eliminate the influence of the electron beam on the spreading dynamics, we repeated the [M_2_C_3_min][Tf_2_N] spreading experiment at a different accelerating voltage of 5.0 kV and confirmed the conclusion that the precursor liquid increases with the first power of time (${\rm{\Delta }}L\ \sim\ {\rm{\Delta }}T$) (Supplementary Figs S5 and S6, and Supplementary Video S3). Further, in order to test the generality of such a conclusion, three similar *in situ* imaging spreading experiments were carried out using three other ionic liquids with different physicochemical properties [34] (Supplementary Table S1), including [C_4_min][Tf_2_N] (Supplementary Fig. S7 and Supplementary Video S4), [C_4_min][TFA] (Supplementary Fig. S8 and Supplementary Video S5) and [C_4_min][TFMS] (Supplementary Fig. S9 and Supplementary Video S6) at an accelerating voltage of 10.0 kV. After dimensionless treatments [23], their spreading processes approximated nearly one straight line (Fig. 2d) and the conclusion that the precursor liquid in the corner spreads linearly over time (${\rm{\Delta }}L\ \sim\ {\rm{\Delta }}T$) was consistently verified. Besides, we also performed the [M_2_C_3_min][Tf_2_N] spreading experiment on the plasma-treated corner. It was exhibited that the precursor liquid still spread linearly with time (Supplementary Fig. S10), illustrating that the linear relationship is independent of the surface chemistry. Therefore, a model describing the dynamic spreading process of the precursor liquid in the corner was established (Fig. 2a).

### Characteristic width of ionic liquid precursors

**Figure 3.**
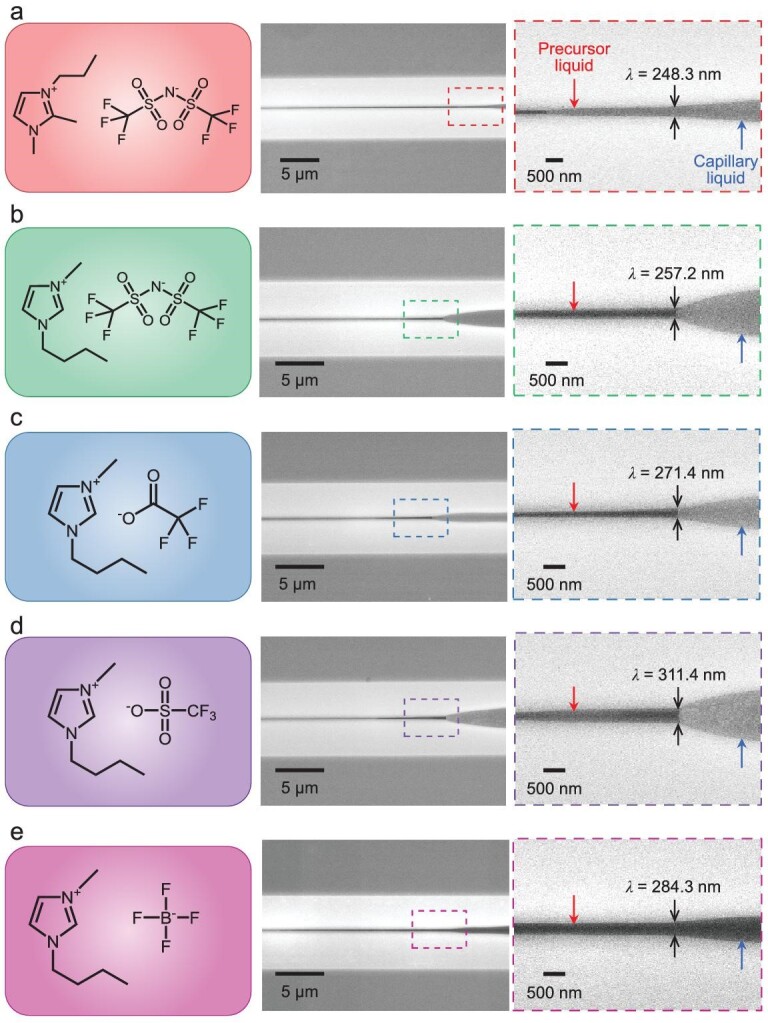
Characteristic widths of precursor liquids for five ionic liquids in a corner. (a–e) SEM images of the five ionic liquids in a corner containing both precursor liquids and capillary liquids, respectively for (a) [M_2_C_3_min][Tf_2_N], (b) 1-butyl-3-methylimidazolium bis(trifluoromethylsulfonyl)imide [C_4_min][Tf_2_N], (c) 1-butyl-3-methylimidazolium trifluoroacetate [C_4_min][TFA], (d) 1-butyl-3-methylimidazolium trifluoromethanesulfonate [C_4_min][TFMS] and (e) 1-butyl-3-methylimidazolium tetrafluoroborate [C_4_min][BF_4_]. The precursor liquids and the capillary liquids are marked with red and blue arrows, respectively. The characteristic widths are marked with black arrow pairs.

As marked in Fig. 2a, the top width of the precursor liquid in the model was defined as the characteristic width [27] ($\lambda $). High-resolution SEM images of the precursor liquids of five spreading ionic liquids (Fig. 3a–e) were captured by virtue of the property of ionic liquids that can be solidified in the ‘reduced display’ mode after concentrated exposure of an electron beam [35]. In the low magnification images (middles of Fig. 3a–e), the precursor liquids can be identified vaguely. After *in situ* magnifications, the frontiers and boundaries of these precursor liquids can be clearly distinguished and their characteristic widths ($\lambda $) were measured. It was found that their $\lambda $ values were all distributed in the range of ∼250–310 nm. Specifically, the $\lambda $ values of [M_2_C_3_min][Tf_2_N] (Fig. 3a), [C_4_min][Tf_2_N] (Fig. 3b), [C_4_min][TFA] (Fig. 3c), [C_4_min][TFMS] (Fig. 3d) and [C_4_min][BF_4_] (Fig. 3e) were 248.3, 257.2, 271.4, 311.4 and 284.3 nm, respectively. Contrastively, the high-resolution SEM images of [M_2_C_3_min][Tf_2_N] precursor liquid at an accelerating voltage of 5.0 kV were also captured and the measured $\lambda $ value was ∼234.1 nm (Supplementary Fig. S11), which was close to that at a 10.0-kV voltage, further supporting the conclusion that the effect of the electron beam on the precursor liquid is neglectable.

**Figure 4.**
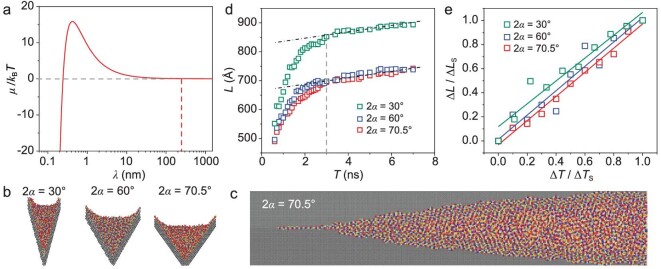
Theoretical calculation and MD simulations of spreading ionic liquid precursors in a corner. (a) Semilogarithmic plot of the chemical potential of [M_2_C_3_min][Tf_2_N] as a function of the liquid top width in the corner with a 2*α* of 70.5°. (b) Section view of the MD simulation models with different 2*α* values. The right sides of the corners with 2*α* of 30° and 60° and both sides of the corner with 2*α* of 70.5° are the (111) crystal face. (c) A snapshot from Supplementary Video 9 illustrating the simulated spreading ionic liquids in the corner with a 2*α* of 70.5°. (d) Simulated real-time spatial–temporal curves of the spreading ionic liquid precursors in corners with different 2*α* values. (e) Dimensionless spatial–temporal plots of the simulated spreading ionic liquid precursors in corners with different 2*α* values. ${\rm{\Delta }}L$ and ${\rm{\Delta }}T$ represent the changes in length and time of precursor liquids after 3 ns, respectively. Δ*L*_s_ and Δ*T*_s_ represent the changes in length and time within the statistical period, respectively.

The generation of the uniform precursor liquid with a characteristic width ($\lambda $) of ∼250–310 nm is accredited to the competing effect of disjoining pressure and Young–Laplace pressure. The disjoining pressure induced by the van der Waals force between the liquids and the corner is the driving factor for reducing the chemical potential. According to Derjaguin approximation [36] (Supplementary Note 1 and Supplementary Fig. S12), the generated disjoining pressure (${{\pi }}$) on the liquids in the corner scales as:


(1) \begin{eqnarray*}{{\pi \ }} &=& \ - 2[\tan \alpha + \tan \left( {90 - 2\alpha } \right)]\\ &&\frac{A}{{12\pi {\lambda }^3{{\cos }}^2\alpha }}\cos \left( {90 - \alpha } \right),\end{eqnarray*}

where *A* is the Hamaker constant, and *L* and $\lambda $ are the length and top width of the liquids in the corner, respectively. At the same time, the generation of the precursor liquid is resisted by the Young–Laplace pressure $P\ = \ 2\gamma /\lambda $, where$\ \gamma $ is the surface tension of the liquids. These two pressures competitively give rise to the chemical potential of the liquids in the corner ($\mu $):


(2) \begin{eqnarray*}\mu &=& \frac{{2\gamma \Omega }}{\lambda } - 2\Omega [\tan \alpha + \tan \left( {90 - 2\alpha } \right)]\\ &&\frac{A}{{12\pi {\lambda }^3{{\cos }}^2\alpha }}\cos \left( {90 - \alpha } \right),\end{eqnarray*}

where $\Omega $ is the molecular volume of the liquid. The semilogarithmic plot of the chemical potential of ionic liquids in corner ($\mu $) as a function of the top width of the liquids ($\lambda $), with [M_2_C_3_min][Tf_2_N] as a representative, is presented as the red line in Fig. 4a. It was shown that the ionic liquids encounter a huge potential barrier when $\lambda $ is <248.3 nm. However, it should be noted that the interpretation of the characteristic width is still in the qualitative stage (Supplementary Fig. S13), especially for precursor liquids in corners with different surface chemistries.

### Universality of ionic liquid precursor spreading dynamics

To gain an in-depth insight into the dynamics of precursor liquids in corners with different opening angles ($2\alpha $), molecular dynamics (MD) simulations were performed to investigate the dynamic spreading process of ionic liquids in silicon corners (Supplementary Note 2). The corner model in the MD simulation was built using silicon atoms (Fig. 4b). An idealized four-site coarse-grained model was built to simulate the molecules of ionic liquids [37] (Supplementary Fig. S14), with a [C_4_min][BF_4_] molecule as a typical example. The ionic liquids spreading process was first simulated on a silicon corner model in which two silicon (111) crystal faces intersected. It was shown that a uniform precursor liquid would be generated in front of the capillary liquid (Fig. 4c and Supplementary Video S9), which is consistent with the experimental results. In order to further prove the universality of the spatial–temporal characteristic of precursor liquids, two other silicon corners with $2\alpha $ of 30° and 60° were constructed to compare the spreading processes of precursor liquids. It was illustrated that the spreading speed of the precursor liquid increases with the decrease in $2\alpha $ values (Fig. 4d). Notably, after 3 ns, the precursor liquid in corners with $2\alpha $ of 30° (Supplementary Video S7), 60° (Supplementary Video S8) and 70.5° (Supplementary Video S9) all forms and spreads linearly over time (Fig. 4d). After measuring the spreading distances of the precursor liquids and dimensionless treatments, it was shown that their spatial–temporal curves were distributed in lines with the same slope (Fig. 4e), which verifies that the spatial–temporal characteristic of the spreading precursor liquid (${\rm{\Delta }}L\ \sim\ {\rm{\Delta }}T$) is universal for corners with different $2\alpha $ values.

## CONCLUSION

In summary, we found that spreading ionic liquid precursors in corners can be directly observed by using *in situ* SEM and established a complete model to describe the dynamic spreading process of liquids in corners—that is, the precursor liquid spreads linearly over time (${\rm{\Delta }}L\ \sim\ {\rm{\Delta }}T$) with a characteristic width of ∼250–310 nm while the capillary liquid follows a power law of $l\sim{t}^{1/2}$. Our results not only provide direct experimental evidence for the spatial–temporal characteristic of precursor liquids, but also may provide a platform for observing other interface phenomena and improving their performance on the microscopic level, including nanofluids, growth of nanomaterials, electrochemical reactions and so on.

## METHODS

### Fabrication of the silicon wafer with parallel corners

Silicon wafers (<100> oriented, N doped, 2∼4 Ω · cm in electrical resistivity, 400 μm in thickness and 10 cm in diameter) were first treated with processes of thermal oxidation and subsequent low-pressure chemical vapor deposition (LPCVP) of Si_3_N_4_ to fabricate a dense oxide and nitridation barrier layer with a thickness of ∼400 nm on their surfaces. Second, the predesigned parallel stripe pattern was transferred onto the silicon wafer surface using photolithography. Then, the exposed oxide and nitridation barrier layer was etched by using reactive-ion etching. After washing off the solidified photoresist using acetone, the silicon wafers were etched with potassium hydroxide solution until the reaction stopped automatically. Finally, the residual barrier layer was removed using hydrogen fluoride solution to produce the corners that we used. The silicon corners were cleaned by using ultrasound with water and ethanol for spreading tests.

### Fabrication of the corners with different opening angles

First, the toothed aluminum alloy molds with different angles were fabricated by using machining. Second, the mold was fluorinated with 1H,1H,2H,2H-perfluorodecyltriethoxysilane. Finally, the polydimethylsiloxane (PDMS) corners with different opening angles were fabricated by replication from these molds. The PDMS corners were exposed to oxygen plasma for 60 s and then were utilized for spreading tests of water and ionic liquids.

### Instruments and characterization

All the *in situ* SEM imaging experiments were carried out using a Hitachi SU8010. The CAs of 2 μL of ionic liquids on silicon surfaces were measured using a contact-angle system (OCA 20, DataPhysics, Germany) and obtained by averaging three CAs at different positions on one sample. Spreading processes of capillary liquids were recorded using a camera (Nikon D7200) after dripping a 2-μL water or ionic liquid droplet into the corners.

## Supplementary Material

nwad119_Supplemental_Files
