# Supplementary material for: Direct observation of spreading precursor liquids in a corner
Source: Natl Sci Rev. 2023 May 5;10(7):nwad119. doi: 10.1093/nsr/nwad119 (PMC10232046; doi:10.1093/nsr/nwad119)
Supplement: nwad119_Supplemental_Files [file nwad119_supplemental_files.zip › Supplementary data.pdf]

SUPPLEMENTARY INFORMATION

Direct observation of spreading precursor liquids in corner

Weining Miao[1,3], Shihao Tian[2,4], Quanzi Yuan[2,4*], Ye Tian[1,3*], Lei Jiang[1,3*]

[1]Key Laboratory of Bioinspired Smart Interfacial Science, Technical Institute of Physics and Chemistry, Chinese Academy of Sciences, Beijing 100190, People's Republic of China.

[2]State Key Laboratory of Nonlinear Mechanics, Institute of Mechanics, Chinese Academy of Sciences, Beijing 100190, People's Republic of China.

[3]School of Future Technology, University of Chinese Academy of Sciences, Beijing 100049, People's Republic of China.

[4]School of Engineering Science, University of Chinese Academy of Sciences, Beijing 100049, People's Republic of China.

*Corresponding author. Email: yuanquanzi@lnm.imech.ac.cn (Q.Y.); tianyely@iccas.ac.cn (Y.T.); jianglei@iccas.ac.cn (L.J.)

This PDF file includes:

Supplementary Note 1-2
Captions for Videos S1-S9
Figures S1-S14
Tables S1-S2

Supplementary Note 1. Calculation of the chemical potential of liquids in corner

When liquids spread along the corner, they will feel the disjoining pressure induced by van der Waals force and Young-Laplace pressure at the same time [1, 2]. First, we consider the van der Waals attraction force between the liquids and the corner.

Basically, the van der Waals attraction force between two planes (F_{vdW}) is given by:

$$F_{\mathrm{vdW}} = \frac{A}{6\pi d^3} \tag{S1}$$

where A is the Hamaker constant and d is the distance between the two planes. In our calculation model, the liquids in the corner are simplified as an inverted isosceles triangle with an apex angle of 2α and a top edge of λ (Fig. S12b) for simplicity. According to the Derjaguin approximation [3], the van der Waals force between the triangle liquids and a plane (F_{P}, Fig. S12a) can be calculated according to:

$$F_{\mathrm{P}} = \int F_{vdW} \frac{\mathrm{d}S}{\mathrm{d}x} \mathrm{d}x \tag{S2}$$

where x is the distance between the plane with a hypothetical plane (the dashed line in Fig. S12a), and S is the cross-sectional area of the hypothetical plane passing through the triangle liquids. Due to $x = \lambda \cos \alpha$, F_{P} is deduced as:

$$F_{\mathrm{P}} = -[\tan \alpha + \tan(90 - 2\alpha)] L \frac{A}{12\pi \lambda^2 \cos^2 \alpha} \tag{S3}$$

where L is the length of the liquids in corner. Therefore, the van der Waals force between the triangle liquids and the corner composed of two intersecting planes (F_{C}, Fig. S12b) can be deduced as:

$$F_{\mathrm{C}} = -2[\tan \alpha + \tan(90 - 2\alpha)] L \cos(90 - \alpha) \frac{A}{12\pi \lambda^2 \cos^2 \alpha} \tag{S4}$$

F_{C} exerts a disjoining pressure (Π) on the liquid in the corner, which is expressed as:

$$\Pi = -2[\tan \alpha + \tan(90 - 2\alpha)] \cos(90 - \alpha) \frac{A}{12\pi \lambda^3 \cos^2 \alpha} \tag{S5}$$

Meanwhile, the behavior of liquids is also controlled by the Young-Laplace pressure:

$$P = \frac{2\gamma}{\lambda} \tag{S6}$$

where γ is the surface tension of the liquids. These two pressures compete with each other and give rise to the chemical potential of the liquids in the corner (μ):

$$\mu = \frac{2\gamma\Omega}{\lambda} - 2\Omega[\tan \alpha + \tan(90 - 2\alpha)] \cos(90 - \alpha) \frac{A}{12\pi \lambda^3 \cos^2 \alpha} \tag{S7}$$

where Ω is the molecular volume of the liquids. Divided by $k_{\mathrm{B}}T$, where k_{B} is the Boltzmann constant and T is the temperature (298 K adopted in the paper), the plot of chemical potentials ($\mu/k_{\mathrm{B}}T$) of ionic liquids, with $[M_2C_3min][Tf_2N]$ as a representative, as a function of the top width of liquids (λ) was shown in Fig. 4a.

Supplementary Note 2. Molecular dynamics (MD) simulation

Large-scale MD simulations were carried out to explore the spreading processes of ionic liquids in the silicon corner using the LAMMPS code [4]. The simulation domain was illustrated in Fig. 4c: ionic liquids spread along the silicon interior corner in a simulation box of 20 × 80 × 20 nm^3. The solid substrate with a corner of 70.52° is composed of 103563 silicon atoms arranged in a cubic crystal structure, which is consistent with the etched silicon corner, proceeding in the ⟨100⟩ direction (into the slice) until the etching front hits the (111) planes. For the models of 30° and 60°, we cut the original lattice to obtain the corner with one surface aligned with the (111) planes. An idealized four-site ionic liquids model was introduced to describe the ionic liquids [C_4min][BF_4] [5-7]. The coarse-grained structure was given in Fig. S14.

The total potential energy E_{ij} between two particles i and j separated by r_{ij} is composed of the short-range van der Waals force energy and the long-range Coulomb interaction, which are respectively calculated by the Lennard-Jones (L-J) potential function and Coulomb law, as follows:

$$E_{ij} = 4\varepsilon_{ij}\left[\left(\frac{\sigma_{ij}}{r_{ij}}\right)^{12} - \left(\frac{\sigma_{ij}}{r_{ij}}\right)^{6}\right] + k_e\frac{q_i q_j}{r_{ij}} \quad \text{(S8)}$$

where ε is the depth of the L-J potential well, σ is the zero-crossing distance for the potential, k_e is the Coulomb constant, and q is the charge of particles. The cut-off lengths for both L-J potential and Coulomb interaction are both 1 nm. All the interaction parameters of the models were shown in Table S2. The values of σ and ε between two different particles were calculated according to the Lorentz–Berthelot rule: $\sigma_{ij} = (\sigma_{ii} + \sigma_{jj})/2$ and $\varepsilon_{ij} = \sqrt{\varepsilon_{ii} * \varepsilon_{jj}}$. The NVT ensemble (constant number of atoms N, volume V, and temperature T) was employed. The Nosé-Hoover thermostat with a time-step of 1 fs was used to regulate the temperature at 350 K.

Videos S1-S9

Video S1. Water spreading process in the silicon corner. The top width of the corner is 70.5 μm and the spacing between two adjacent corners is 180.0 μm.

Video S2. [M_2C_3min][Tf_2N] spreading process in the corner. The accelerating voltage and current are 10.0 kV and 10 μA, respectively.

Video S3. [M_2C_3min][Tf_2N] spreading process in the corner. The accelerating voltage and current are 5.0 kV and 10 μA, respectively.

Video S4. [C_4min][Tf_2N] spreading process in the corner. The accelerating voltage and current are 10.0 kV and 10 μA, respectively.

Video S5. [C_4min][TFA] spreading process in the corner. The accelerating voltage and current are 10.0 kV and 10 μA, respectively.

Video S6. [C_4min][TFMS] spreading process in the corner. The accelerating voltage and current are 10.0 kV and 10 μA, respectively.

Video S7. MD simulated [C_4min][BF_4] spreading process in the corner with 2α of 30°. The right side of the corner is the (111) crystal face.

Video S8. MD simulated [C_4min][BF_4] spreading process in the corner with 2α of 60°. The right side of the corner is the (111) crystal face.

Video S9. MD simulated [C_4min][BF_4] spreading process in the corner with 2α of 70.5°. Both sides of the corner are the (111) crystal face.

Supplementary Figures

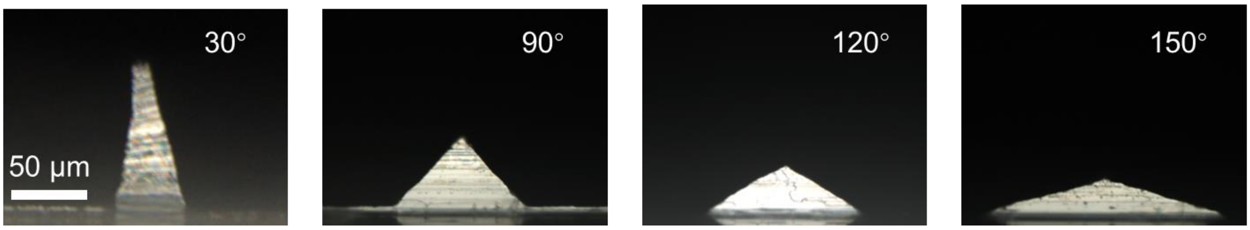

Figure S1. The toothed aluminum alloy molds with different angles. The dentate structures have the same cross-sectional area of 1750 μm^2 for ensuring the same flux.

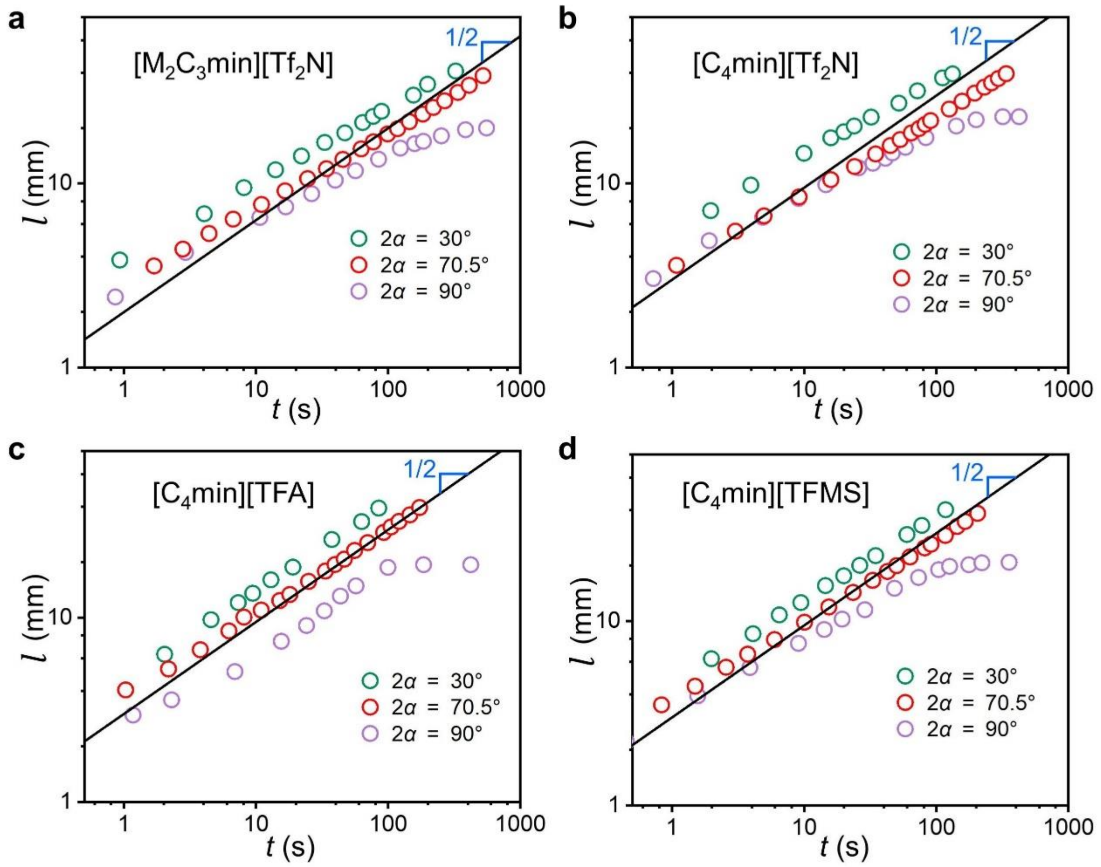

Figure S2. Logarithmic plots of the spreading length of capillary liquids of four ionic liquids versus time. They all follow a power law of 1/2.

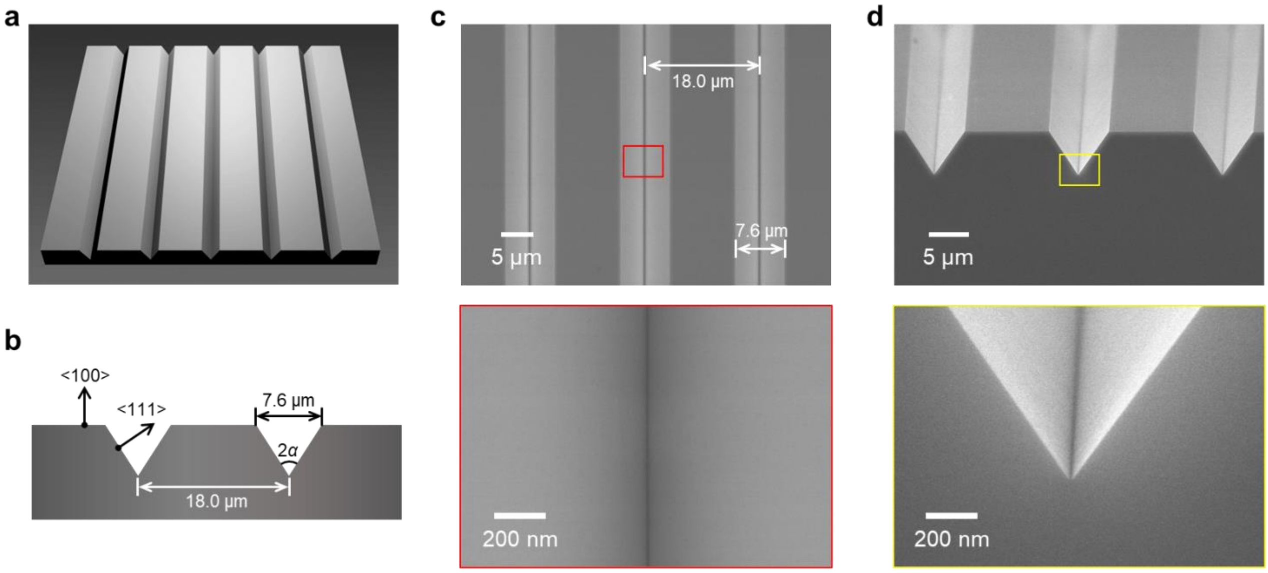

Figure S3. The fabricated silicon wafer with parallel corners. (a,b) Schematic of a 2 cm × 2 cm silicon wafer containing parallel corners. The corners are fabricated by anisotropic etching two intersecting silicon (111) crystal faces on a <100> silicon wafer with potassium hydroxide. The opening angle (2α) of the corner is about 70.5°. (c,d) Top-view and section-view SEM images of the parallel corners. The top width of the corner is 7.6 μm and the spacing between two adjacent corners is 18.0 μm. The surfaces are smooth and clean.

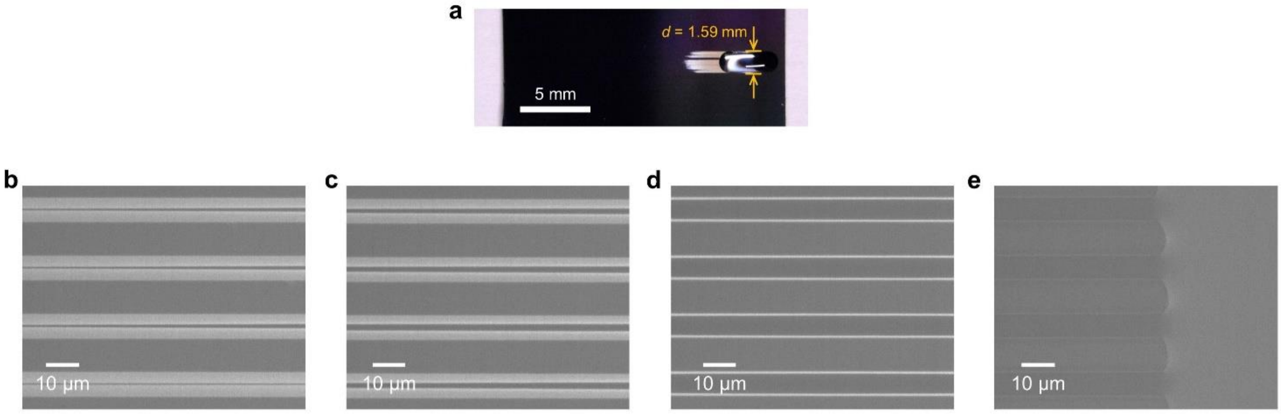

Figure S4. Optical image and full view SEM images of spreading [M_2C_3min][Tf_2N] in the corners. (a) Optical image of 1 μL [M_2C_3min][Tf_2N] on silicon corner. The volume of the droplet is much larger than the corner. Limited by the imaging field size of SEM, we cannot obtain a complete overview of the spreading liquid in the parallel corners. Hence, SEM images at four typical positions from the frontier of spreading [M_2C_3min][Tf_2N] precursor liquid to the macroscopic liquid are exhibited from (b) to (e) in sequence. The bulk liquids are on the right side of the meniscuses in (e), and the states of the other five ionic liquids are similar to [M_2C_3min][Tf_2N].

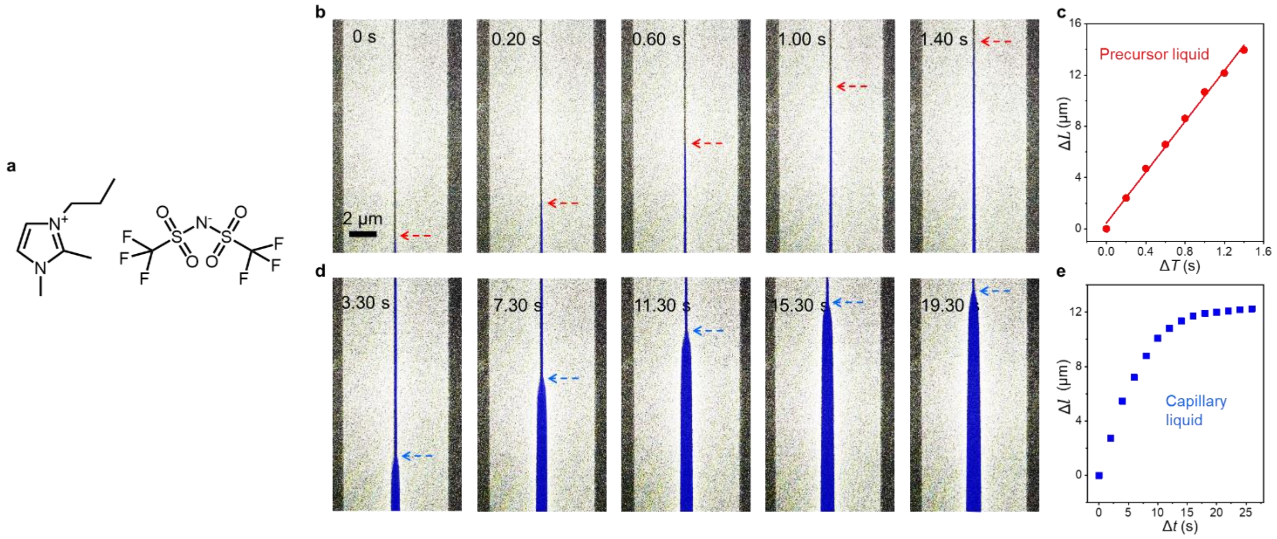

Figure S5. Spreading processes of [M_2C_3min][Tf_2N] under 5.0 kV accelerated electron beam. (a) Molecular formula of the ionic liquids [M_2C_3min][Tf_2N]. (b,d) SEM snapshots of the spreading precursor liquid and capillary liquid captured from [M_2C_3min][Tf_2N] spreading video (Video S3) under a 5.0 kV accelerated electron beam, respectively. The liquids are pseudo-colored in blue, and the forefront positions of precursor liquid and capillary liquid are marked with dashed red and blue arrows, respectively. (c,e) Real-time temporal-spatial characteristics of the spreading precursor liquid and capillary liquid, respectively. [M_2C_3min][Tf_2N] precursor liquid and capillary liquid spread with the first power of time and the square root of time under a 5 kV accelerated electron beam, respectively. $\Delta t = 0$ in (e) represents 3.30 s in (d).

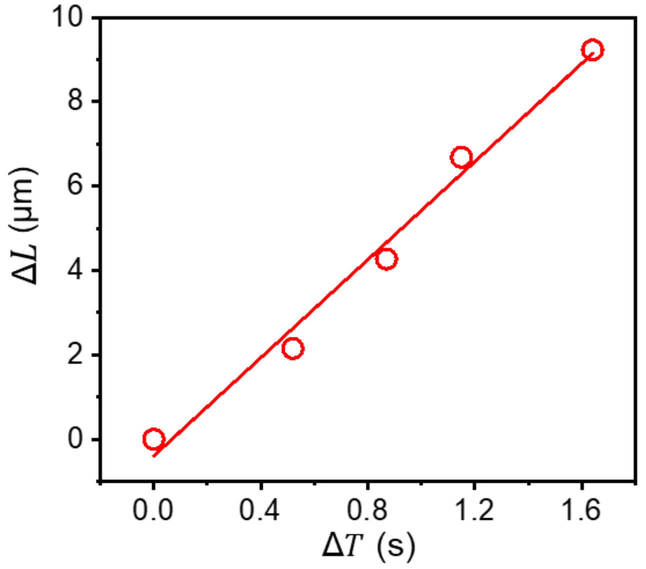

Figure S6. Real-time temporal-spatial characteristics of the spreading [M_2C_3min][Tf_2N] precursor liquid under 5.0 kV accelerated electron beam at the later stage of the spreading process. The precursor liquid spread linearly over time with a relatively slower speed, which should be attributed to the restriction of the spreading speed of capillary liquid (Fig. S2).

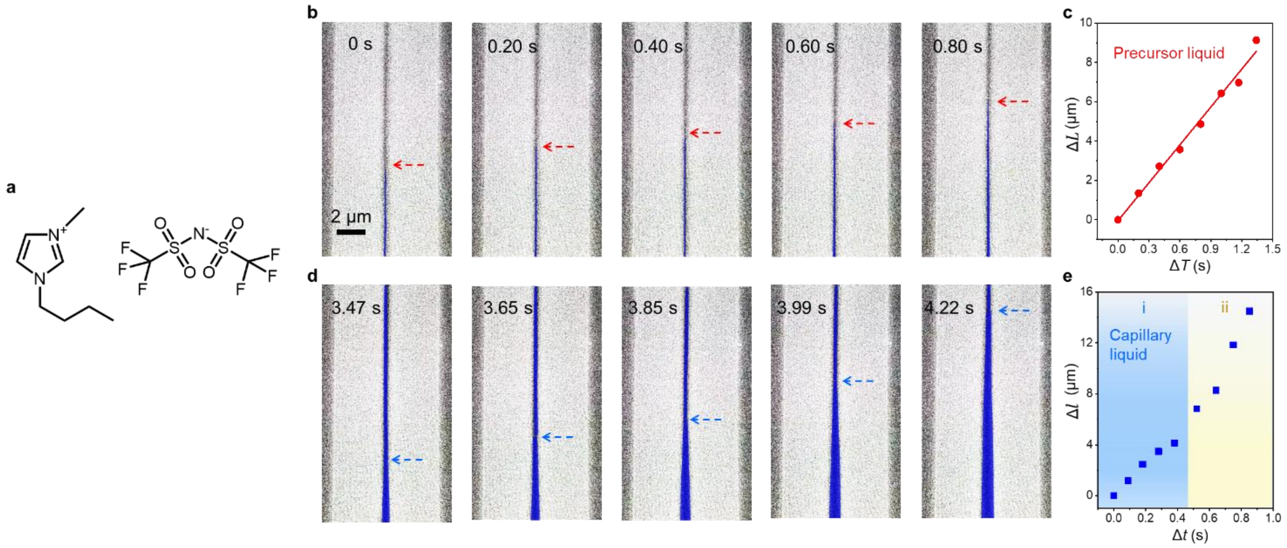

Figure S7. Spreading processes of [C_4min][Tf_2N] under 10.0 kV accelerated electron beam. (a) Molecular formula of [C_4min][Tf_2N]. (b,d) SEM snapshots of the spreading precursor liquid and capillary liquid captured from [C_4min][Tf_2N] spreading video (Video S4), respectively. (c,e) Changes of forefront position of precursor liquid and capillary liquid versus time, respectively. [C_4min][Tf_2N] precursor liquid spread with the first power of time. $\Delta t = 0$ in (e) represents 3.47 s in (d). The different spreading speeds in stage i and ii of capillary liquid in (e) was resulted from the rise in temperature.

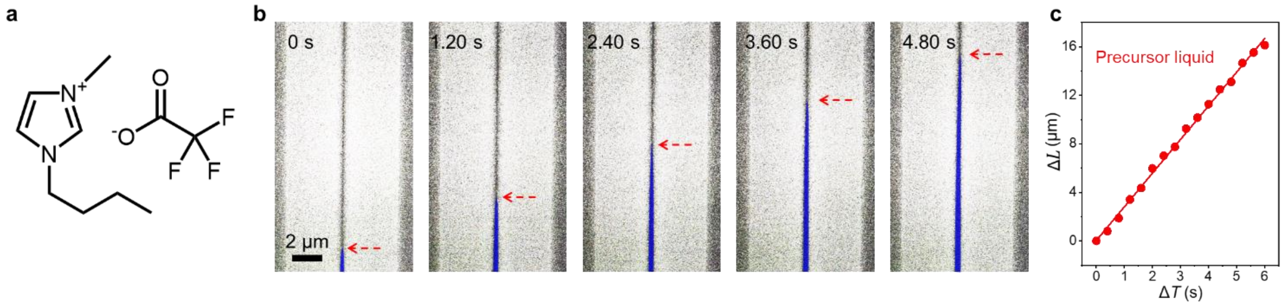

Figure S8. Spreading processes of [C_4min][TFA] under 10.0 kV accelerated electron beam. (a) Molecular formula of [C_4min][TFA]. (b) SEM snapshots of the spreading precursor liquid captured from [C_4min][TFA] spreading video (Video S5). (c) Real-time temporal-spatial characteristic of the spreading precursor liquid. [C_4min][TFA] precursor liquid spreads linearly with time. Since the boundary between the precursor liquid and capillary liquid of [C_4min][TFA] is not easy to be distinguished, the forefront positions of capillary liquids are not measured.

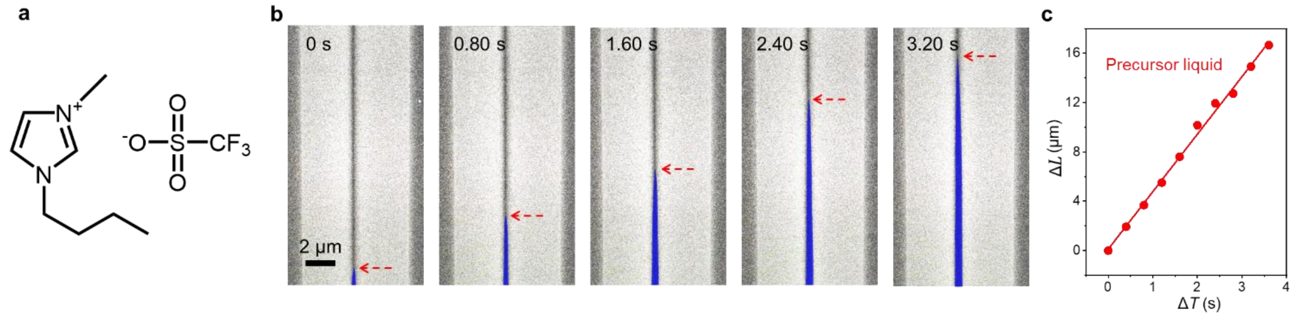

Figure S9. Spreading processes of [C_4min][TFMS] under 10.0 kV accelerated electron beam. (a) Molecular formula of [C_4min][TFMS]. (b) SEM snapshots of the spreading precursor liquid captured from [C_4min][TFA] spreading video (Video S6). (c) Real-time temporal-spatial characteristic of the spreading precursor liquid. [C_4min][TFMS] precursor liquid spreads linearly with time. Since the boundary between the precursor liquid and capillary liquid of [C_4min][TFMS] is not easy to be distinguished, the forefront positions of capillary liquids are not measured.

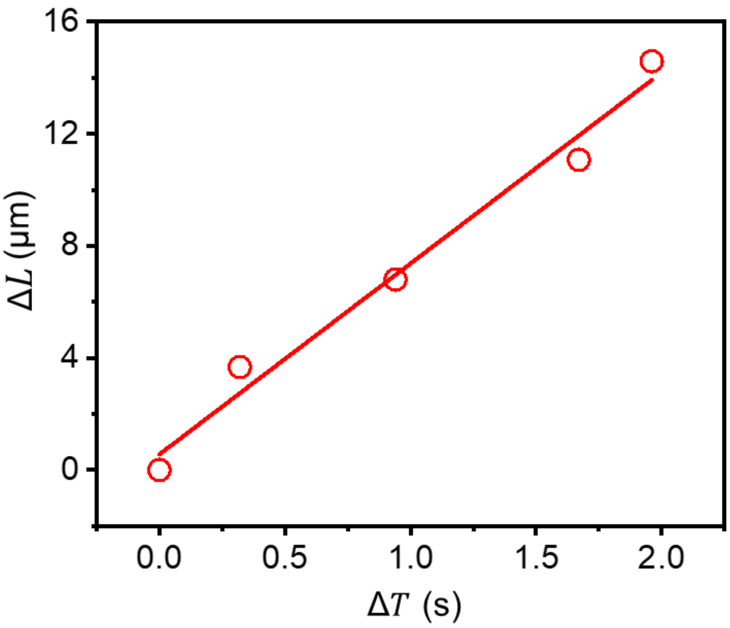

Figure S10. Spreading processes of [M_2C_3min][Tf_2N] precursor liquid in plasma-treated silicon corner under 5.0 kV accelerated electron beam.

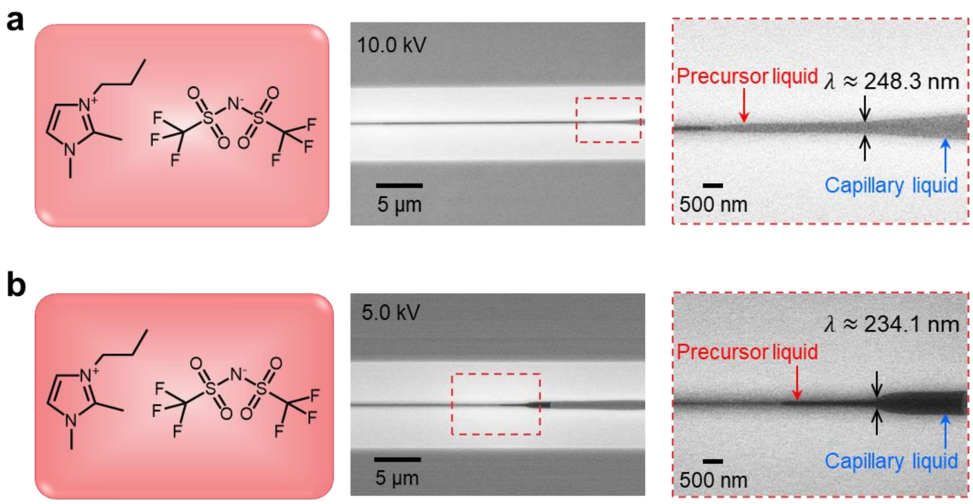

Figure S11. SEM images of [M_2C_3min][Tf_2N] in corner containing both precursor liquid and capillary liquid. The characteristic widths (λ) of [M_2C_3min][Tf_2N] precursor liquid at 10.0 kV (a) and 5.0 kV (b) are close and are respectively 248.3 nm and 234.1 nm, illustrating that the effect of the electron beam on ionic liquids precursor is neglectable.

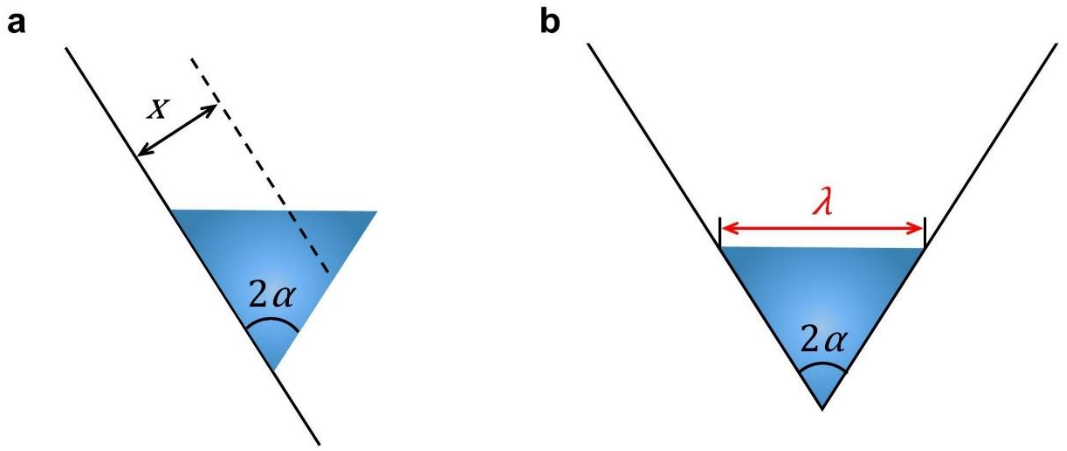

Figure S12. Models for the derivation of van der Waals attraction force between liquids and the corner. (a) Model for calculating the van der Waals attraction force between the inverted isosceles triangle-shaped liquid with an apex angle of 2α and a plane. (b) Model for the precursor liquid wetting a corner. The top width of the triangle-shaped liquid is defined as λ.

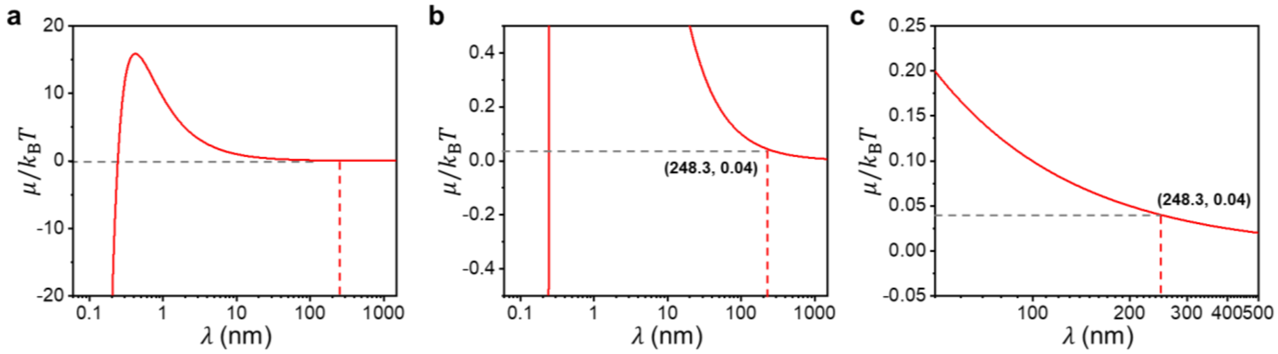

Figure S13. Full and enlarged semilogarithmic plots of the chemical potential of $[M_2C_3min][Tf_2N]$as a function of the liquid top width in the corner. The location of the characteristic width is marked. Interestingly, the chemical potential of $[M_2C_3min][Tf_2N]$at its characteristic width of 248.3 nm was about 0.04, which is the same value where precursor liquid appears in Ref [8].

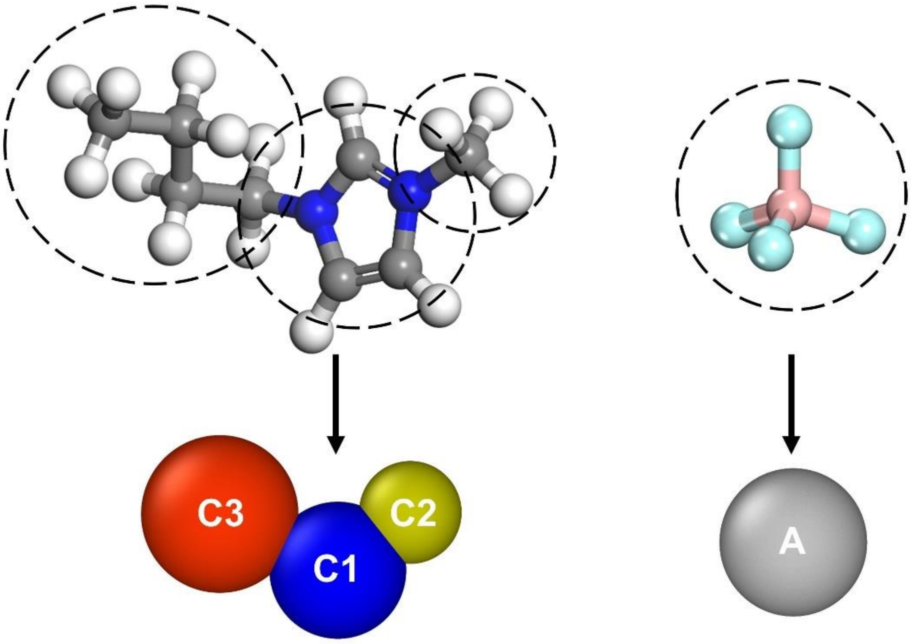

Figure S14. Coarse-grained model of [C_4min][BF_4]. C1, C2, and C3 represent the imidazole ring, methyl chain, and butyl chain in the cation, respectively, and A represents the anion.

Supplementary Tables

Table S1. Viscosity, surface tension [9, 10], and intrinsic contact angle (CA) data for ionic liquids.

Ionic liquids	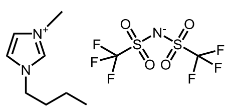 [C_4min][Tf_2N]	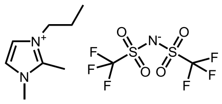 [M_2C_3min][Tf_2N]	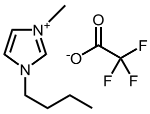 [C_4min][TFA]	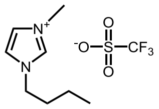 [C_4min][TFMS]	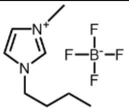 [C_4min][BF_4]
Viscosity (cP)	52	90	73	90	219
Surface tension (dyn/cm)	37.5	41.0	43.9	31.9	46.6
CA (°)	23.2 ±0.7 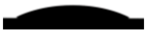	27.7 ±0.7 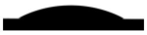	32.7 ±0.6 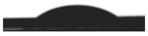	23.0 ±1.2 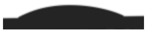	36.7 ±0.1 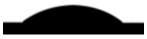

Table S2. Parameters of cationic and anionic coarse particles and other atoms in MD simulations [5].

Interaction site	x (Å)	y (Å)	z (Å)	M (g/mol)	σ_{ii} (Å)	ε_{ii} (kcal/mol)	q_i (e)
A	0.000	0.000	0.000	86.81	4.51	0.6116	-0.7800
C1	0.000	-0.527	1.365	67.07	4.38	0.0860	0.4374
C2	0.000	1.641	2.987	15.04	3.41	0.4372	0.1578
C3	0.000	0.187	-2.389	57.12	5.04	0.7740	0.1848
Si	-	-	-	28.09	3.39	0.6186	0.0000
O	-	-	-	16.00	3.17	0.1554	-0.8476
H	-	-	-	1.01	0.00	0.0000	0.4238

References

1. de Gennes PG. Wetting: Statics and dynamics. *Rev Mod Phys* 1985; **57**: 827-863.
2. Bonn D, Eggers J, Indekeu J *et al.* Wetting and spreading. *Rev Mod Phys* 2009; **81**: 739-805.
3. Derjaguin B. Analysis of friction and adhesion iv the theory of the adhesion of small particles. *Kolloid-Zeitschrift* 1934; **69**: 155-164.
4. Thompson AP, Aktulga HM, Berger R *et al.* Lammps-a flexible simulation tool for particle-based materials modeling at the atomic, meso, and continuum scales. *Comput Phys Commun* 2022; **271**: 108171.
5. Merlet C, Salanne M, Rotenberg B. New coarse-grained models of imidazolium ionic liquids for bulk and interfacial molecular simulations. *J Phys Chem C* 2012; **116**: 7687-7693.
6. Song FH, Ma B, Fan J *et al.* Molecular dynamics simulation on the electrowetting behaviors of the ionic liquid bmim bf4 on a solid substrate. *Langmuir* 2019; **35**: 9753-9760.
7. Roy D, Patel N, Conte S *et al.* Dynamics in an idealized ionic liquid model. *J Phys Chem B* 2010; **114**: 8410-8424.
8. Huang J, Lo YC, Niu JJ *et al.* Nanowire liquid pumps. *Nat Nanotechnol* 2013; **8**: 277-281.
9. Huddleston JG, Visser AE, Reichert WM *et al.* Characterization and comparison of hydrophilic and hydrophobic room temperature ionic liquids incorporating the imidazolium cation. *Green Chem* 2001; **3**: 156-164.
10. Hu X, Zhang S, Qu C *et al.* Ionic liquid based variable focus lenses. *Soft Matter* 2011; **7**: 5941-5943.
